# Validation of continuous clinical indices of cardiometabolic risk in a cohort of Australian adults

**DOI:** 10.1186/1471-2261-14-27

**Published:** 2014-02-27

**Authors:** Suzanne J Carroll, Catherine Paquet, Natasha J Howard, Robert J Adams, Anne W Taylor, Mark Daniel

**Affiliations:** 1 Spatial Epidemiology and Evaluation Research Group, School of Population Health and Sansom Institute for Health Research, University of South Australia, Adelaide, South Australia, Australia; 2 Research Centre of the Douglas Mental Health University Institute, Verdun, Québec, Canada; 3 Discipline of Medicine, The University of Adelaide, Adelaide, South Australia, Australia; 4 Department of Medicine, The University of Melbourne, St. Vincent’s Hospital, Melbourne, VIC, Australia

**Keywords:** Cardiometabolic, Cardiovascular disease, Type 2 diabetes, Risk scores, ROC, AUC, Validation

## Abstract

**Background:**

Indicators of cardiometabolic risk typically include non-clinical factors (e.g., smoking). While the incorporation of non-clinical factors can improve absolute risk prediction, it is impossible to study the contribution of non-clinical factors when they are both predictors and part of the outcome measure. Metabolic syndrome, incorporating only clinical measures, seems a solution yet provides no information on risk severity. The aims of this study were: 1) to construct two continuous clinical indices of cardiometabolic risk (cCICRs), and assess their accuracy in predicting 10-year incident cardiovascular disease and/or type 2 diabetes; and 2) to compare the predictive accuracies of these cCICRs with existing risk indicators that incorporate non-clinical factors (Framingham Risk Scores).

**Methods:**

Data from a population-based biomedical cohort (n = 4056) were used to construct two cCICRs from waist circumference, mean arteriole pressure, fasting glucose, triglycerides and high density lipoprotein: 1) the mean of standardised risk factors (cCICR-Z); and 2) the weighted mean of the two first principal components from principal component analysis (cCICR-PCA). The predictive accuracies of the two cCICRs and the Framingham Risk Scores were assessed and compared using ROC curves.

**Results:**

Both cCICRs demonstrated moderate accuracy (AUCs 0.72 – 0.76) in predicting incident cardiovascular disease and/or type 2 diabetes, among men and women. There were no significant differences between the predictive accuracies of the cCICRs and the Framingham Risk Scores.

**Conclusions:**

cCICRs may be useful in research investigating associations between non-clinical factors and health by providing suitable alternatives to current risk indicators which include non-clinical factors.

## Background

Various indicators of cardiometabolic risk are accepted for use in population health studies. Well-known examples include the Framingham Risk Scores for cardiovascular disease (CVD) and type 2 diabetes. While such indicators can have both clinical and research utility, composite indicators of risk typically include non-clinical factors such as behaviour (e.g., smoking in the Framingham 10-year General CVD Risk Score), family history (e.g., ASSIGN, Framingham Diabetes Risk Score), or area-level disadvantage (e.g., ASSIGN, QRISK) [1-4]. The inclusion of these non-clinical factors in composite outcomes usually improves absolute risk prediction. This approach however, can have limitations for inferential research.

For instance, in studies that evaluate the mechanisms by which area-level characteristics (e.g., area-level disadvantage) influence cardiometabolic risk, non-clinical factors are often framed as mediators and thus must be excluded from composite expressions of cardiometabolic risk that are evaluated as outcomes [5]. A variable cannot be both a predictor and an outcome. Adapting established indicators of cardiometabolic risk by removing non-clinical components can reduce the utility of such risk indicators and necessitate re-validation. Composite measures incorporating only clinical factors to express cardiometabolic risk are required to support the investigation of associations between cardiometabolic risk and sociodemographic and behavioural factors.

One composite indicator of cardiometabolic risk based solely on clinical risk factors is metabolic syndrome. Current definitions of metabolic syndrome incorporate counts of risk factors exceeding established threshold values wherein individuals are classified as either having, or not having the syndrome [6,7]. However, the underlying expression of each risk measure is continuous, and cardiometabolic risk a progressive function of these combined risk measures [8-10]. Using current methods for defining metabolic syndrome, for example the International Diabetes Federation (IDF) and Adult Treatment Panel III (ATPIII) definitions [6,7], a minimal change in risk measures can result in a change of classification status [10], yet a large change may not. A continuous index of risk constructed from clinical factors would eliminate this issue and more closely approximate an individual’s actual continuum of risk, providing information relating to risk severity. Evidence supports the use of a continuous clinical index of cardiometabolic risk (cCICR) in health studies [11,12] and the use of such a measure has been recommended by the American Diabetes Association and the European Association for the Study of Diabetes [8].

Different methods of constructing a cCICR, typically from metabolic syndrome components, have been employed. These cCICRs include: a count of risk factors exceeding recommended thresholds [13]; the sum of risk factor points established by risk factor deciles [14]; the sum or mean of z-scores [11,12,15]; and components derived by principal component analysis [16,17]. While incorporating a count of risk factors improves the utility of metabolic syndrome status as an expression of risk, this strategy still does not account for the progressive nature of risk within each risk factor. Using deciles to express each risk factor is a progressive approach but still compromises statistical power and distributional information due to categorisation [18]. Arguably, the most progressive and efficient cCICRs are those involving the summation of z-scores and principal component analysis derived scores. A cCICR constructed as the mean of standardised metabolic syndrome components has been validated for use in children and adolescents [11,15], though not in adult populations. However, an alternative method utilising principal component analysis has been validated for use in adult populations [16,17]. No study thus far has constructed such cCICRs for use in adult populations and evaluated their validity. The aims of this study were: 1) to construct two cCICRs and assess their accuracy in predicting 10-year incident CVD and/or type 2 diabetes in a longitudinal adult cohort; and 2) to compare these cCICR s with existing risk indicators comprising clinical, sociodemographic and behavioural factors (Framingham CVD [F-CVD] and Diabetes [F-T2DM] Scores).

## Methods

This research study was part of the Place and Metabolic Syndrome (PAMS) Project which aims to assess the mechanisms that explain the relationships between place, health behaviour and cardiometabolic health. The PAMS Project received ethical approval from the University of South Australia, Central Northern Adelaide Health Service, Queen Elizabeth Hospital, and South Australian Department for Health and Aging Human Research Ethics Committees.

### Participants

The North West Adelaide Health study (NWAHS), a 10-year biomedical cohort comprised of 4056 randomly selected men and women aged 18 years and over, provided data for this study. The NWAHS has involved three waves of data collection thus far, Wave 1 (2000–03, n = 4056), Wave 2 (2005–06, n = 3563), and Wave 3 (2008–10, n = 2871). Each wave has involved the collection of standardised measures using Computer-Assisted Telephone Interviews, self-report paper questionnaires, and clinic visits. Written informed consent was obtained from all participants prior to each wave of data collection. Further information on recruitment has previously been published [19].

### Measures

#### Biomedical data

Biomedical data were collected during the hospital-based clinic visits at each wave. Measures included blood pressure (mean of two measures), height and weight, waist and hip circumference (mean of three measures), and a fasting blood sample which was used to determine triglyceride, total cholesterol, high density lipoprotein (HDL), glucose and glycosylated haemoglobin (HbA1c) concentrations. Participants were asked about previously diagnosed type 2 diabetes, heart attack, stroke, angina, or transient ischaemic attack/mini stroke. Further details of data collection are available elsewhere [19].

### Risk scores

Risk factor measures used to construct cCICRs were transformed (log 10) to improve their distribution. The five factors used in the construction of the cCICRs were: waist circumference, mean arteriole pressure (MAP = [2DBP + SBP]/3), triglycerides, fasting blood glucose, and HDL (multiplied by −1 to account for its protective effect). These risk factors have previously been used to construct cCICRs [13,15] and represent cardiometabolic risk components used to define metabolic syndrome [6,7]. Each risk factor was sex-standardised to account for sex-related differences.

#### Mean of z-scores (cCICR-Z)

A cCICR was constructed as the mean of the standardised risk factors. A higher score signifies greater cardiometabolic risk compared to the sample population.

#### Principal component analysis (cCICR-PCA)

A second cCICR was constructed using principal component analysis (orthogonal rotation). Principal component analysis was performed using the five standardised risk factors, stratified by sex. The risk score, cCICR-PCA, was constructed using the weighted sum of the first two principal components with the proportion of variance explained as weights following the methods of Wijndaele and colleagues [16]. These first two components explained a total of 61.69% of the variance (41.86% and 19.83%; Eigenvalues 2.09 and 0.99) among men, and 60.14% of the variance (42.75% and 18.39%; Eigenvalues 2.14 and 0.92) among women.

#### Framingham risk scores

General CVD risk (10-year risk) and type 2 diabetes risk (8-year risk) were calculated using the relevant Framingham Risk Score algorithm [1,3].

#### Incident type 2 diabetes and cardiovascular disease

Participants were considered to have type 2 diabetes if they had HbA1c values ≥ 6.5% (48 mmol/mol) [20], fasting plasma glucose level ≥ 7 mmol/L, or self-reported previous diagnosis by a doctor. CVD was determined from self-reported doctor diagnosis only. Incident CVD was coded for participants who were CVD-free at baseline but determined to have CVD at Wave 2 or 3. Incident type 2 diabetes was similarly coded. A third outcome measure, cardiometabolic disease, was constructed by combining incident CVD and type 2 diabetes.

### Analyses

The ability of the risk scores to predict 10-year incident CVD and/or type 2 diabetes was assessed using odds ratios, true positive rate, false positive rate, receiver operating characteristics (ROC) curves, and the area under the curve (AUC). True positive rate (i.e., sensitivity) is the probability of a positive test outcome for a diseased individual while false positive rate (i.e., 1-specificity) is the probability of a positive test outcome for a non-diseased individual. A perfect risk score would have a true positive rate of 1 and false positive rate of 0 [21].

Odds ratios were calculated to provide a measure of the strength of association between indicators of risk and incident CVD and/or type 2 diabetes. For metabolic syndrome, odds ratios were calculated using two-by-two frequencies tables. For the continuous risk scores (cCICRs and Framingham Risk Scores), odds ratios were calculated using sex-specific age-adjusted logistic regression models predicting each outcome. Continuous risk scores were standardised (by sex) prior to analysis to allow for comparisons of odds ratios across regression models.

As a strong association (e.g., a large odds ratio) between outcome and predictor does not necessarily imply good predictive accuracy in correctly classifying an individual [21], ROC curves were therefore generated by plotting true positive rate by false positive rate across the possible range of values for each risk score. The AUCs were then calculated, providing a measure of predictive accuracy. An AUC of 0.5 represents prediction equal to chance, an AUC of 1.0, perfect prediction [22].

Differences between the predictive accuracies of the cCICRs and the Framingham Risk Scores were assessed by statistically comparing the AUCs. For comparison purposes, true positive rate for each of the risk scores was calculated by fixing false positive rate at the levels for metabolic syndrome (ATPIII definition [7]) in predicting CVD and/or type 2 diabetes. False positive rate for each score was similarly calculated. All analyses were conducted in STATA (version 12.1, StataCorp, Texas, USA).

## Results

At baseline, 1898 men and 2095 women (total n = 3993) had complete clinical measures and information on CVD and type 2 diabetes status (Table 1). Men demonstrated worse profiles than women in regard to weight, waist girth, proportion overweight, glucose, triglycerides, HDL, blood pressure measures, and the proportion identified as having metabolic syndrome, type 2 diabetes, and CVD. Women demonstrated a worse profile than men in regard to proportion obese.

**Table 1:** Baseline characteristics of the sample according to sex

**Characteristic**	**Men (n = 1898)**	**Women (n = 2095)**	**P value**	**Total (n = 3993)**
Age (years)	50.80 (16.76)	50.20 (16.05)	0.25	50.49 (16.39)
Weight (kg)	85.32 (15.98)	72.25 (15.74)	<0.0001	78.46 (17.38)
Waist girth (cm)	98.43 (13.03)	87.09 (14.12)	<0.0001	92.47 (14.74)
BMI (kg/m^2^)	27.89 (4.78)	27.75 (6.04)	0.41	27.82 (5.48)
Overweight (BMI 25.00 – 29.99 kg/m^2^)^1^	874 (46.05%)	663 (31.65%)	<0.0001	1537 (38.49%)
Obese (BMI ≥ 30 kg/m^2^)^1^	512 (26.98%)	632 (30.17%)	0.03	1144 (28.65%)
Glucose (mmol/L)^2^	5.1 (4.8-5.6)	4.9 (4.5-5.3)	<0.0001	5.00 (4.6-5.5)
Triglycerides (mmol/L)^2^	1.3 (0.9-2.0)	1.1 (0.8-1.6)	<0.0001	1.20 (0.9-1.8)
HDL cholesterol (mmol/L)^2^	1.2 (1.0-1.4)	1.5 (1.2-1.7)	<0.0001	1.30 (1.1-1.6)
Total cholesterol (mmol/L)^2^	5.1 (4.4-5.8)	5.2 (4.6-6.0)	0.0001	5.2 (4.5-5.9)
Systolic blood pressure (mmHg)	130.90 (17.61)	125.50 (19.15)	<0.0001	128.03 (18.63)
Diastolic blood pressure (mmHg)	82.65 (9.96)	78.79 (10.04)	<0.0001	80.63 (10.19)
Mean arteriole pressure (mmHg)	98.72 (11.20)	94.35 (11.89)	<0.0001	96.43 (11.77)
Metabolic syndrome (ATPIII)^1^	553 (29.14%)	461 (22.00%)	<0.0001	1014 (25.39%)
Type 2 diabetes^1^	218 (11.49%)	170 (8.11%)	0.0003	388 (9.72%)
Cardiovascular disease^1^	199 (10.48%)	120 (5.73%)	<0.0001	319 (7.99%)
Cardiometabolic disease^1^	363 (19.13%)	251 (11.98%)	<0.0001	614 (15.38%)

Mean (SD) *t*-test; ^1^proportions and Chi square; ^2^median (Q1-Q3) and Wilcoxon Signed Rank.

*Abbreviations*: *BMI* Body mass index, *ATPIII* Adult Treatment Panel III definition.

**Figure 1:**
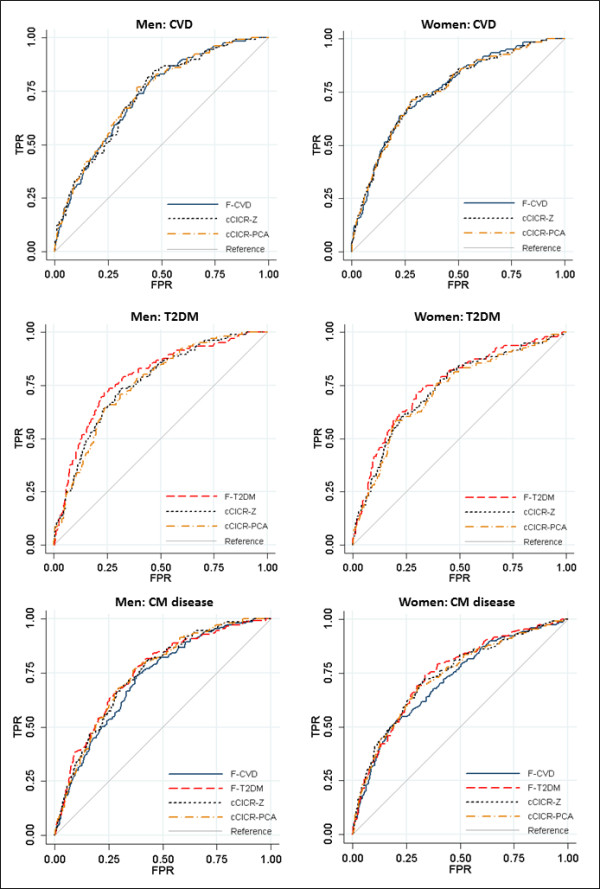
ROC curves for models predicting CVD, type 2 diabetes (T2DM) and cardiometabolic (CM) disease among men and women.

Sample loss and sample size used for models predicting the different outcomes are shown in Table 2. The AUCs, true positive rates, false positive rates, and odds ratios for each of the risk scores in predicting 10-year incident disease are shown in Table 3. As metabolic syndrome status is categorical, direct comparison of the odds ratios for metabolic syndrome status and the continuous risk scores in predicting cardiometabolic outcomes is not possible.ROC curves are shown in Figure 1. Both cCICRs demonstrated significant associations (odds ratios) with incident CVD among men and women, and AUCs for both models reflect moderate predictive accuracy. In both men and women, the F-CVD demonstrated moderate predictive accuracy and was not significantly different to the cCICRs in this regard. These similarities in predictive accuracies were reflected in the true positive rate and false positive rate for the different scores with similar values for true positive rates at the fixed false positive rate of 0.27 (the rate for metabolic syndrome), and similar false positive rates with true positive rate fixed at 0.41.

**Table 2:** Sample loss and sample used in models predicting each outcome among men and women

	**Men**			**Women**		
	**CVD**	**T2DM**	**CM disease**	**CVD**	**T2DM**	**CM disease**
Disease free at baseline	1699	1680	1535	1975	1925	1844
Loss due to no 10-year incident data	563	648	603	618	722	717
Loss due to incomplete FRS data	12	98	97	9	113	103
Total sample	1124	934	835	1348	1091	1024
Incidence	130	106	153	122	96	159
Incidence rate (%)	11.57	11.35	18.32	9.05	8.80	15.53

*Abbreviations*: *CVD* Cardiovascular disease, *T2DM* Type 2 diabetes, *CM* Cardiometabolic, *FRS* Framingham Risk Score.

**Table 3:** **Associations and predictive accuracies (95**% **CIs) for indicators of risk predicting disease incidence (age-adjusted models)**

	**CVD**				**T2DM**				**CM disease**			
	**AUC**	**TPR**^**1**^	**FPR**^**2**^	**OR**	**AUC**	**TPR**^**1**^	**FPR**^**2**^	**OR**	**AUC**	**TPR**^**1**^	**FPR**^**2**^	**OR**
**Men**	**n = 1124**				**n = 934**				**n = 835**			
MetS ^3^	-	0.41	0.27	1.90	-	0.52	0.22	3.72	-	0.42	0.21	2.69
		(0.32–0.50)	(0.24–0.29)	(1.31–2.78)		(0.42–0.61)	(0.20–0.25)	(2.46–5.63)		(0.34–0.50)	(0.18–0.24)	(1.86–3.89)
cCICR-Z	0.73	0.54	0.16	1.62	0.76	0.59	0.17	2.48	0.75	0.50	0.15	2.03
	(0.69–0.77)	(0.44–0.64)	(0.08–0.24)	(1.33–1.99)	(0.71–0.81)	(0.49–0.70)	(0.11–0.23)	(1.90–3.25)	(0.71–0.79)	(0.40–0.59)	(0.10–0.20)	(1.62–2.55)
cCICR-PCA	0.73	0.59	0.15	1.65	0.75	0.60	0.20	2.32	0.75	0.54	0.16	2.01
	(0.69–0.78)	(0.49–0.69)	(0.09–0.22)	(1.35–2.03)	(0.71–0.80)	(0.48–0.73)	(0.16–0.25)	(1.79–3.00)	(0.71–0.79)	(0.44–0.63)	(0.12–0.20)	(1.61–2.51)
F-CVD	0.73	0.55	0.16	2.45	-	-	-	-	0.72	0.49	0.16	2.71
	(0.69–0.77)	(0.46–0.65)	(0.10–0.22)	(1.63–3.68)					(0.68–0.77)	(0.40–0.58)	(0.11–0.22)	(1.78–4.13)
F-T2DM	-	-	-	-	0.79	0.68	0.15	2.27	0.75	0.54	0.15	1.81
					(0.74–0.83)	(0.57–0.79)	(0.09–0.20)	(1.84–2.80)	(0.71–0.79)	(0.45–0.64)	(0.08–0.21)	(1.51–2.16)
**Women**	**n = 1348**				**n = 1091**				**n = 1024**			
MetS ^3^	-	0.27	0.19	1.54	-	0.40	0.13	4.21	-	0.28	0.13	2.68
		(0.19–0.36)	(0.17–0.22)	(1.01–2.35)		(0.30–0.49)	(0.11–0.16)	(2.69–6.58)		(0.21–0.34)	(0.11–0.15)	(1.80–3.99)
cCICR-Z	0.76	0.57	0.06	1.24	0.73	0.40	0.14	2.04	0.74	0.43	0.06	1.66
	(0.71–0.80)	(0.47–0.66)	(0.03–0.09)	(1.03–1.51)	(0.68–0.79)	(0.28–0.52)	(0.09–0.19)	(1.62–2.58)	(0.69–0.78)	(0.35–0.52)	(0.03–0.09)	(1.37–2.02)
cCICR–PCA	0.76	0.58	0.06	1.28	0.73	0.35	0.14	1.97	0.73	0.43	0.06	1.63
	(0.72–0.80)	(0.49–0.68)	(0.02–0.09)	(1.05–1.56)	(0.67–0.78)	(0.24–0.47)	(0.09–0.19)	(1.56–2.48)	(0.69–0.78)	(0.34–0.52)	(0.04–0.09)	(1.34–1.97)
F-CVD	0.76	0.56	0.08	1.59	-	-	-	-	0.72	0.42	0.08	1.84
	(0.72–0.80)	(0.46–0.65)	(0.05–0.10)	(1.07–2.35)					(0.67–0.76)	(0.32–0.51)	(0.05–0.11)	(1.25–2.69)
F-T2DM	-	-	-	-	0.75	0.46	0.09	2.00	0.74	0.41	0.07	1.63
					(0.70–0.81)	(0.35–0.56)	(0.05–0.14)	(1.65–2.42)	(0.70–0.78)	(0.32–0.50)	(0.04–0.11)	(1.38–1.93)

^1^TPR with FPR fixed at MetS levels; ^2^FPR with TPR fixed at MetS levels; ^3^not age-adjusted.

*Abbreviations*: *CVD* Cardiovascular disease, *T2DM* Type 2 diabetes, *CM* Cardiometabolic, *AUC* Area under the curve, *TPR* True positive rate, *FPR* False positive rate, *OR* Odds ratio, *MetS* Metabolic syndrome, *cCICR-Z* Continuous clinical index of cardiometabolic risk constructed as the mean of standardised risk scores, *cCICR-PCA* Continuous clinical index of cardiometabolic risk constructed using principal component analysis, *F-CVD* Framingham 10-year General CVD Risk Score, *FT2DM* Framingham 8-year Diabetes Risk Score.

For type 2 diabetes, the cCICRs were significantly associated with disease incidence in both sexes, and demonstrated moderate predictive accuracies. Higher odds ratios were evident between the cCICRs and incident type 2 diabetes than for incident CVD. The F-T2DM also demonstrated moderate predictive accuracy. The true positive rate for F-T2DM was slightly higher, and false positive rate slightly lower than the rates for the cCICRs. This was reflected in the slightly higher AUCs in predicting incident type 2 diabetes among both men and women. However, the difference in AUCs between models was not significant.

Both cCICRs were significantly associated with incident cardiometabolic disease among men and women, again demonstrating moderate predictive accuracies. Similarly, the F-CVD and F-T2DM demonstrated moderate predictive accuracies. There were no statistically significant differences in predictive accuracies of the risk scores in predicting incident cardiometabolic disease among men or women. The true positive rates and false positive rates were again similar for each of the risk scores for both sexes.

## Discussion

The objective of this study was to validate two cCICRs, developed to address limitations of dichotomous risk measures such as metabolic syndrome, and continuous indicators of cardiometabolic risk that include non-clinical factors such as behaviour. This study constructed two cCICRs that had previously been validated in either children or adult populations, and assessed their accuracies in predicting 10-year incident CVD and/or type 2 diabetes. The predictive accuracies of the cCICRs were then compared with established risk scores that incorporate sociodemographic and behavioural factors.

The two cCICRs constructed for use in this study demonstrated statistically significant associations with 10-year incident CVD, type 2 diabetes and cardiometabolic disease among men and women. Although the odds ratios for men were higher than for women for all outcomes, post-hoc analyses revealed that these differences were not statistically significantly different (p-values > 0.05).

Previous studies have assessed associations between a cCICR constructed as the first principal component of principal component analysis and incident CVD and type 2 diabetes. In one study among men and women (44–84 years) the first principal component demonstrated a hazard ratio of 1.65 (1.49-1.82) in models predicting 5.5-year CVD events, adjusted for age, sex and ethnicity [10]. Another study using the first principal component in sex-specific age-adjusted models demonstrated odds ratios of 3.4 (2.6-4.4) and 5.1 (3.6-7.2) for men and women (30–65 years) respectively in predicting 9-year incident type 2 diabetes – stronger associations than those found in the current study [17]. Associations were weaker in predicting 9-year incident CVD, with estimated odds ratios being 1.7 (1.4-2.1) and 1.7 (1.0-2.7) for men and women respectively, similar to the levels of association found in the current study. This pattern of stronger associations when predicting type 2 diabetes to weaker associations with CVD is not unexpected. Studies have found clinical indicators of risk, including metabolic syndrome status and a count of metabolic syndrome risk components, to have stronger associations with incident type 2 diabetes than incident CVD [23,24].

The differences in the strengths of associations in predicting incident type 2 diabetes between this study and that of Hillier and colleagues [17] may be due to differences in the age range of study participants, the current study including individuals as young as 18 years. Neither previous study reported information on model fit or predictive accuracy. No study has yet examined the association and accuracy of a cCICR constructed as a mean of standardised risk factors in predicting incident CVD and/or type 2 diabetes in adults.

Both cCICRs demonstrated moderate accuracy in predicting incident CVD, type 2 diabetes and cardiometabolic disease, performing similarly well to the Framingham Risk Scores. These findings are interesting as the F-CVD and F-T2DM both include other well established risk factors in their construction. However, differences in the strength of association are noted for the F-CVD in predicting CVD and may be attributed to the inclusion of smoking status in the risk algorithm.

The two cCICRs constructed in this study performed similarly well to the Framingham Risk Scores and thus constitute viable expressions of cardiometabolic risk. Furthermore, these cCICRs can be used to represent a combined CVD and type 2 diabetes risk (i.e., cardiometabolic risk) though associations are stronger for type 2 diabetes alone. As such, cCICRs may be useful in research studies that require a cardiometabolic risk measure that is constructed solely from clinical measures.

This study found little difference in the performance of the two differently constructed cCICRs. Therefore, the choice of which measure to use within a study may depend on the design and intent of the study. The cCICR-Z places equal weight on each of the risk factors included in its construction though each of these factors may have a greater or lesser association with cardiometabolic risk. However, this does not appear to have negatively impacted the index’s performance when compared to cCICR-PCA which weights the components based on a principal component analysis variance matrix. This may, however, make the cCICR-Z the more useful measure for tracking change in risk over time. Unlike the cCICR-PCA, the weightings of the components that are used to construct the cCICR-Z will not change each time the score is constructed, for example, at each time point in a longitudinal study.

A number of limitations of this study should be considered. This study made use of self-reported CVD status; type 2 diabetes status was determined based on combined self-reported status and clinical measures. However, any effect this may have had on the strengths of associations and predictive accuracies of the indicators in predicting incident CVD and type 2 diabetes will apply equally for each of the risk indicators.

The analyses in this study featured age-adjusted models. Other potential risk factors may also affect the associations reported, notably smoking status. The exclusion of models adjusting for such behavioural (and other) factors is intentional as the purpose of this study was to assess the predictive accuracy of indicators of cardiometabolic risk constructed solely from clinical risk factors. Studies intending to use cCICRs can adjust for additional factors as deemed appropriate.

In addition to the above, other clinical risk factors have demonstrated associations with cardiometabolic risk including proinflammatory and prothrombotic markers [8]. The inclusion of such risk factors may improve the accuracies of cCICRs in predicting incident cardiometabolic disease. However, these measures were not available for the current study.

Medication status (being on antihypertensive or lipid-lowering medications) was not considered in the construction and analyses of the cCICRs. Only a small number of participants were taking these medications, providing only small sample sizes for analyses of predictive accuracy. For example, models predicting cardiometabolic disease incidence amongst men taking medications (antihypertensive or lipid-lowering medications) would have included a sample of only 94 individuals. As such analyses stratified by medication status were not performed. However, post-hoc sensitivity analyses excluding individuals on antihypertensive or lipid-lowering medications suggested no notable difference in the AUCs for any of the models (results not reported). This is consistent with results from two studies validating a cCICR constructed using principal component analysis among adults [10,17] which both concluded that patterns of association were similar when performing sensitivity analyses excluding participants taking antihypertensive or lipid-lowering medications.

The cCICRs constructed in this study are sample specific, providing a relative measure of risk, each individual’s risk being compared to the population sample being assessed. The Framingham Risk Scores provide estimates of absolute risk and would be the preferred choice of measures wherever absolute CVD or type 2 diabetes risk is the objective. The relative nature of the cCICRs may limit comparison across studies in different populations. This may also have implications for their use in longitudinal studies where the sample may change over time due to loss to follow-up.

## Conclusions

This study constructed two cCICRs and assessed their associations with, and accuracy in prediction of 10-year incident CVD and/or type 2 diabetes. The predictive accuracies of these indices were compared with Framingham Risk Scores which include clinical, sociodemographic and behavioural factors in their risk algorithms. The cCICRs demonstrated similar abilities to the Framingham Risk Scores in predicting incident CVD, type 2 diabetes, and cardiometabolic disease. Little difference was noted in the performance of the two cCICRs constructed using different methods, however the cCICR-Z may be easier to use and interpret. Either cCICR may be useful in research investigating associations between cardiometabolic risk and sociodemographic or behavioural factors. Both cCICRs provide a suitable alternative to current indicators of risk which either include non-clinical factors or lack information on risk severity.

## Abbreviations

AUC: Area under the curve; CCICR: Continuous clinical index of cardiometabolic risk; cCICR-PCA: A cCICR constructed using principal component analysis; cCICR-Z: A cCICR constructed as the mean of standardised risk scores; CVD: Cardiovascular disease; DBP: Diastolic blood pressure; F-CVD: Framingham 10-year General CVD Risk Score; F-T2DM: Framingham 8-year Diabetes Risk Score; FPR: False positive rate; HbA1c: Glycosylated haemoglobin; HDL: High density lipoprotein; MAP: Mean arteriole pressure; MetS: Metabolic syndrome; NWAHS: North West Adelaide Health Study; OR: Odds ratio; PAMS Project: Place and Metabolic Syndrome Project; ROC: Receiver operating characteristics; SBP: Systolic blood pressure; TPR: True positive rate.

## Competing interests

The authors declare that they have no competing interests.

## Authors’ contributions

SJC, MD, CP, NJH, AWT, and RJA conceived and designed the study. SJC analysed the data under guidance of CP. SJC, CP, MD contributed to interpretation of results. SJC wrote the manuscript, and CP, NJH, and MD revised it critically for important intellectual content. All authors approved the final manuscript.

## Pre-publication history

The pre-publication history for this paper can be accessed here:

http://www.biomedcentral.com/1471-2261/14/27/prepub
